# pH-Potentiometric Investigation towards Chelating Tendencies of *p*-Hydroquinone and Phenol Iminodiacetate Copper(II) Complexes

**DOI:** 10.1155/2010/125717

**Published:** 2010-06-08

**Authors:** Marios Stylianou, Anastasios D. Keramidas, Chryssoula Drouza

**Affiliations:** ^1^Department of Chemistry, University of Cyprus, 1678 Nicosia, Cyprus; ^2^Agricultural Production and Biotechnology and Food Science, Cyprus University of Technology, P. O. Box 50329, 3603 Lemesos, Cyprus

## Abstract

Copper ions in the active sites of several proteins/enzymes interact with phenols and quinones, and this interaction is associated to the reactivity of the enzymes. In this study the speciation of the Cu^2+^ with iminodiacetic phenolate/hydroquinonate ligands has been examined by pH-potentiometry. The results reveal that the iminodiacetic phenol ligand forms mononuclear complexes with Cu^2+^ at acidic and alkaline pHs, and a binuclear O_phenolate_-bridged complex at pH range from 7 to 8.5. The binucleating hydroquinone ligand forms only 2 : 1 metal to ligand complexes in solution. The pK values of the protonation of the phenolate oxygen of the two ligands are reduced about 2 units after complexation with the metal ion and are close to the pK values for the copper-interacting tyrosine phenol oxygen in copper enzymes.

## 1. Introduction

Copper ions in the active sites of proteins/enzymes mediate a broad scope of chemical processes including electron transfer, dioxygen uptake, storage, and transport and catalytic conversions [1]. When surveying the known copper enzymes and their functions, it is striking that their reactivity is typically linked to dioxygen or compounds directly synthesized from O_2_-like phenols and quinones [2–7].

For example, copper proteins are involved in reversible dioxygen binding in hemocyanin [8], two-electron reduction to peroxide coupled to oxidation of substrates in amine and galactose oxidases [9], biogenesis of novel metalloenzyme cofactors (e.g., topaquinone in amine oxidases) [10], activation of hydroxylation in tyrosinase [11], and proton pumping in cytochrome c oxidase [12].

**Scheme 1:**
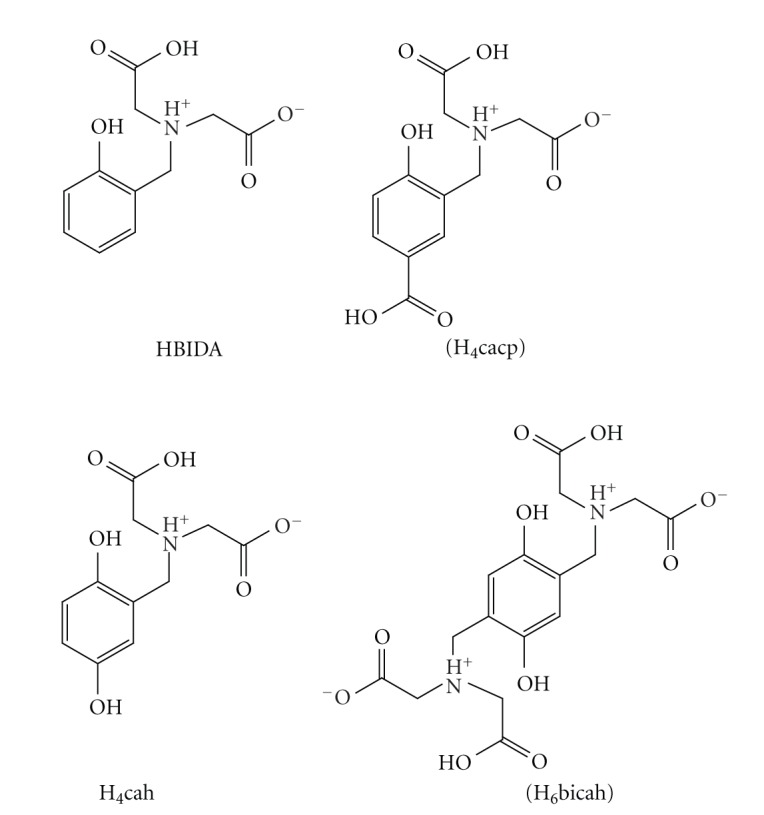
Iminodiacetic derivatives of phenol/*p*-hydroquinone ligands with their abbreviations. The ligands referred to the potentiometric/stability studies are denoted in parentheses.

**Scheme 2:**
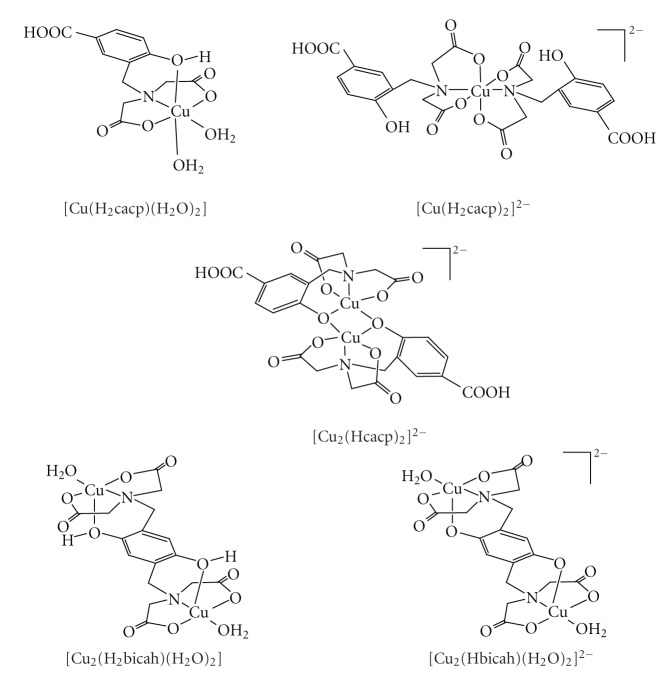
Molecular drawings of the structures of the phenol and *p*-hydroquinone iminodiacetate copper(II) complexes, isolated at a pH range 2.0–9.0 according to a recent detailed crystallographic study [25].

Detailed study of the solid and solution chemistry of Cu^2+^ phenolate/hydroquinonate complexes is essential for better understanding of the coordination of the metal ion in the enzymes and the mechanisms of the enzymatic catalysis. Derivatives of phenol or hydroquinone containing nitrogen [13–22] as donor atoms are the vast majority of the ligands used to model the active site of the copper enzymes. Despite the importance of phenolate/hydroquinonate chelating ligands as models of copper enzymes, ligands with other than nitrogen donor atoms such as aminocarboxylate derivatives of phenols, have been much less studied. These ligands exhibit very attractive features for modelling metal enzymes, such as the highly solubility in aqueous solution, forming stable complexes with metal ions and the similarity of the donor groups to those in biological systems. In addition, the one-electron oxidized *p*-semiquinone radical of the ligand 2,5-bis[N,N-bis(carboxymethyl)aminomethyl] hydroquinone (H_6_bicah) has been stabilized in aqueous solution by ligation to metal ions [23] and thus serves as model for the enzymes that operate via a *p*-semiquinone radical, acting in one-electron transfer reactions, including cytochrome c and copper amine oxidases. In previous pH-potentiometric studies [24] of Cu^2+^ with the phenol iminodiacetate ligand HBIDA (Scheme 1) the equilibrium calculations have been performed assuming that all the species of Cu^2+^ with HBIDA in solution at various pHs are mononuclear 1 : 1 and 1 : 2 metal to ligand complexes. A recent detailed crystallographic study [25] of the Cu^2+^-phenol iminodiacetate H_4_cacp, H_4_cah and H_6_bicah (Scheme 1) complexes isolated at a pH range 2.0–9.0 has shown that binuclear O_phenolate_-bridged Cu^2+^ complexes (Scheme 2) are also present in solution. It is apparent that previous pH-potentiometric studies of these systems should be repeated including also the dinuclear species in the calculations.

Herein, we describe the pH-potentiometric studies of Cu^2+^ with the iminodiacetate phenolate tripod ligands H_4_cacp and H_6_bicah. In contrast to H_4_cacp, H_6_bicah exhibits two metal ion binding sites bridged through the hydroquinone moiety. The potentiometric study showed that only the H_4_cacp ligand forms in solution O_phenolate_-bridged binuclear complexes, which is also in agreement with the previous crystallographic study [25]. The pK values of the protonation of the phenolate oxygen of the two ligands reduced about 2 units after complexation with the metal ion are close to the pK values for the copper-interacting tyrosine phenol oxygen in copper enzymes, such as glyoxal oxidase [26].

## 2. Experimental Section

### 2.1. Materials

Copper(II) acetate monohydrate, *p*-hydroquinone, 4-hydroxybenzoic acid, iminodiacetic acid, paraformaldehyde, potassium chloride, and potassium hydrogen phthalate were obtained from Aldrich. Sodium hydroxide and hydrogen chloride were purchased from Merck. All chemicals were reagent grade and used without further purification.

### 2.2. Ligand Preparation

The ligands referred to this study 2,5-bis[*N*,*N*′-bis(carboxymethyl)aminomethyl]-hydroquinone (H_6_bicah) and 2-[*N*,*N*′-bis(carboxymethyl)aminomethyl]-4-carboxyphenol (H_4_cacp) were synthesized based on the Mannich type reaction reported in the literature [27, 28]. The synthesis of the organic ligands (Scheme 1) was performed under inert nitrogen atmosphere and their purity was checked and confirmed by means of ^1^H-NMR spectroscopy. ^1^H-NMR spectra were recorded on a 300.13 MHz Avance Brucker spectrometer.

### 2.3. Potentiometric Studies and Computational Data Analysis

The potentiometric equilibrium measurements of H_4_cacp and H_6_bicah ligands in the absence and in the presence of metal ions were carried out with a JENWAY 3020 pH meter fitted with an Ag-AgCl reference electrode in saturated KCl solution. A glass electrode was calibrated as a hydrogen concentration probe by titrating known amounts of HCl with CO_2_-free NaOH solution, and the equivalence point was determined by Gran's method which yields the standard potential *E*° of the electrode, using the GLEE computational program [29]. The actual concentration of NaOH (0.157 mol dm^−3^) was standardized by titration with potassium hydrogen phthalate, and the HCl solution (0.111 mol dm^−3^) was standardized by titration of the standard NaOH solution. The temperature was maintained at 298 K and the ionic strength of each experimental sample was adjusted to 0.100 mol dm^−3^ with the addition of KCl-supporting electrolyte. Typical concentrations of experimental solutions were 5.00 mmol dm^−3^ in ligand with molar concentration of copper (II) ion half, equivalent, and twice to that of the ligand. Degassed distilled water was used for the preparation of the solutions and the oxygen and carbon dioxide contamination of the reaction mixtures from the atmosphere was avoided by continuous passing of purified nitrogen gas in the reaction cell. 

The proton association constants of H_4_cacp and H_6_bicah ligands and the formation constants of 1 : 1 (H_4_cacp : Cu^2+^) and 1 : 2 (H_6_bicah : 2Cu^2+^) metal-ligand systems were obtained using the program TIRMET which is a computational program based on mass-balance and charge-balance equations, written in our laboratory according to the basic principles first reported by Martell and Motekaitis [30, 31]. In this program the input consists of the components and their concentrations, the initial values of the equilibrium constants for each species considered to be present, the potentiometric equilibrium data determined experimentally, and conditions of the potentiometric experimental procedure (*E*°, pK_*w*_ = 13.78 at 298 K, *γ* = 0.78). The program sets up simultaneous mass-balance equations for all components at each neutralization value involving the concentration of acid added to the assay and solves for each species present in the pH region 2.00–10.0. Then, equilibrium constants are varied in order to minimize the differences between the calculated and observed values, resulting in the fitting of the calculated results to the experimental curves. The concentration stability constants, *β*_*pqr*_ = [M_*p*_L_*q*_H_*r*_]/[M]^*p*^[L]^*q*^[H]^*r*^, were considered to be estimated according to the model proposed by the computational program PSEQUAD [32]. The species considered present in the assays are those expected to be formed according to established principles of coordination chemistry including the formation of deprotonated and protonated metal chelates, respectively [24, 33–35]. All potentiometric titrations were performed three times for each system (about 100 data points each) in the pH range 2.00–10.0 without significant variation.

## 3. Results and Discussion

### 3.1. Ligands

**Figure 1:**
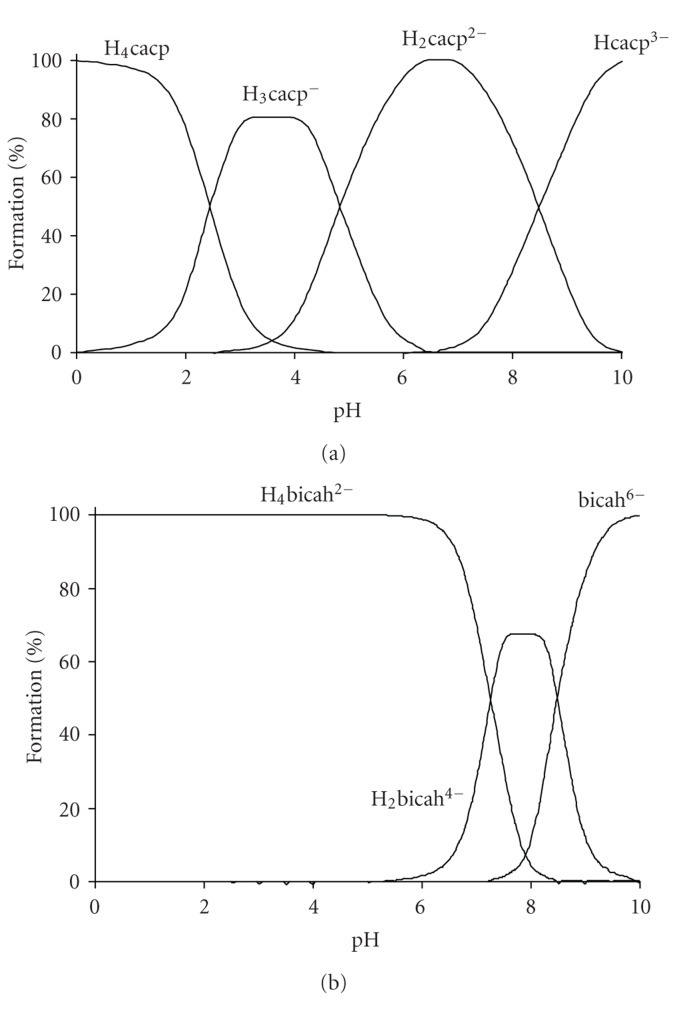
Species distribution (% formation) for the phenol (a) and *p*-hydroquinone (b) iminodiacetate ligands as a function of pH over the range 2.00–10.0 at molar concentration 5.00 mmol dm^−3^ (25°C, *I* = 0.10 mol dm^−3^ KCl, pK_*w*_ = 13.78, and *γ* = 0.78).

 Potentiometric titrations of phenol (H_4_cacp) and *p*-hydroquinone (H_6_bicah) iminodiacetate derivatives indicate stepwise protonation steps arising from their characteristic functional groups, amine, carboxylates, and phenolate, in the measurable pH range. The protonation constants (overall stability protonation constants log  *β*) are listed in Tables 1 and 2, respectively, and their distribution speciation diagrams are illustrated in Figure 1.

The pH-metric titration curve of H_4_cacp indicates three major protonation steps due to the phenolate or the benzoic-carboxylate oxygen group, the carboxylate oxygen group, and the amino group with pK_*a*_ values 8.47, 4.84, and 2.42, respectively (Table 1). The low pK_*a*_ (2.42) value attributed to the amine nitrogen atom demonstrates intramolecular hydrogen bonding between the deprotonated amino group and the phenolic hydrogen. Such bonding stabilizes the deprotonated form of the nitrogen and thus facilitates loss of the hydrogen ion as shown by the lower pK_*a*_ value which is similar to that found for an analogue ligand [*N*-(o-hydroxybenzyl)iminodiacetic acid] [24](HBIDA, Scheme 1) (2.34) while for the nonphenolic, iminodiacetic acid (ida) the corresponded value is 2.94 [33]

The pH-metric titration of the symmetric bis-substituted iminodiacetate *p*-hydroquinone derivative H_6_bicah gave two steps each one corresponding to two successive protonation of the two phenolate oxygens and the two carboxylate groups with pK_*a*_ values 8.47 and 7.26, respectively (Table 2). It was not possible to determine the pK_*a*_ value for the amine nitrogen group because this value was very low.

### 3.2. Cu(II)-*H*_4_cacp

**Figure 2:**
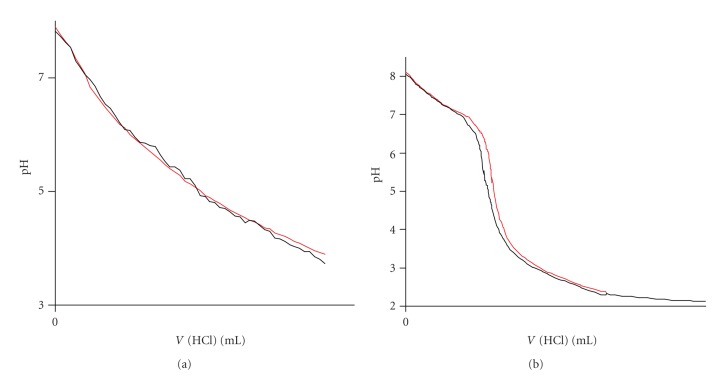
Potentiometric titrations for the Cu(II)-H_4_cacp (1 : 1) and Cu(II)-H_6_bicah (2 : 1) systems (a) and (b), respectively) as a function of pH over the range 2.00–10.0 at molar concentration 2.50 mmol dm^−3^ based on ligand (25°C, *I* = 0.10 mol dm^−3^ KCl, pK_*w*_ = 13.78, *γ* = 0.78, and HCl = 0.111 mmol dm^−3^). The line denoted with black colour refers to the experimental titration curve while the red refers to the computational fitting of the obtained experimental data.

**Figure 3:**
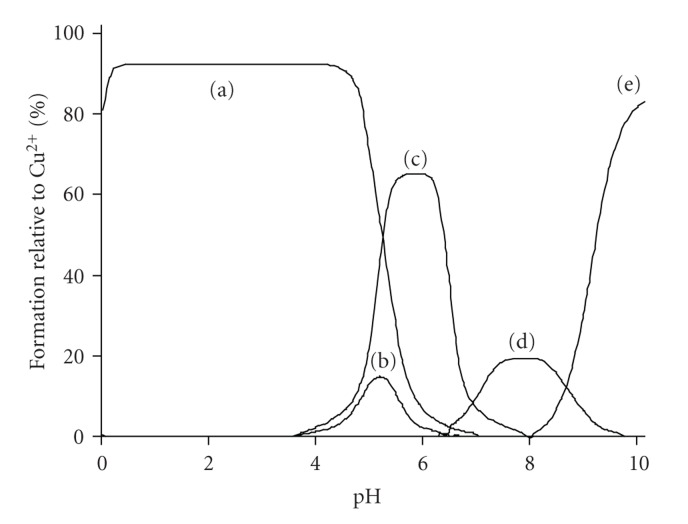
Species distribution (% formation relative to Cu^2+^) in the Cu(II)-H_4_cacp system at equimolar concentration (5.00 mmol dm^−3^) as a function of pH over the range 2.00–10.0 (25°C, *I* = 0.10 mol dm^−3^ KCl, pK_*w*_ = 13.78, *γ* = 0.78). The Cu(II) species are as follows: (a) [Cu(H_2_cacp)(H_2_O)], (b) [Cu(H_2_cacp)_2_]^2−^, (c) [Cu(Hcacp)(H_2_O)]^−^, (d) [Cu_2_(Hcacp)_2_]^2−^, and (e) [Cu(Hcacp)(OH)]^2−^.

The Cu(II)-H_4_cacp titration curves were evaluated on the assumption of the formation of various 1 : 1, 1 : 2 and 2 : 1 metal to ligand species with different protonation steps. The extensive crystallographic study of the isolated complexes from solutions of Cu(II)-H_4_cacp at various pHs reported by Stylianou et al. [25] was also used for the better suggestion of the species in solution (Scheme 2). The best fit with the experimental data (Figure 2(a)) was obtained with the speciation model listed in Table 1. Species distribution curves for the complexes formed in the Cu(II)-H_4_cacp system as a function of pH are depicted in Figure 3. 

Cu(II) ion forms with H_4_cacp three major mononuclear species, the protonated [Cu(H_2_cacp)(H_2_O)] at pH below 5.0, the deprotonated [Cu(Hcacp)(H_2_O)]^−^ at pH between 5.0 and 6.5 the mono-hydroxo species [Cu(Hcacp)(OH)]^2−^ at pH above 9.0 and a minor 1 : 2 metal to ligand [Cu(H_2_cacp)_2_]^2−^ species at pH 5. 

The process from the deprotonated mononuclear species to the protonated one, which corresponds to the consumption of one H^+^ per molecule of complex equation (1), is accompanied by a color change from green to blue attributed to the protonation of the phenolic oxygen. The protonation of the phenolic oxygen will result in weakening or nonbonding of the Cu–OH(phenol) bond which is in agreement with the color change (the mononuclear nonphenolic amino acetate complexes of Cu^2+^ at acidic pHs exhibit blue color). The crystallographic data of the complex isolated at pH 3.2 [25] confirm the weak interaction between the protonated phenol oxygen atom and the metal ion [Cu–OH(phenol), 2.529(2) Å]: 


(1)
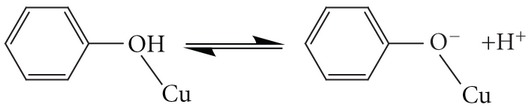



The estimated pK_*a*_ involved in this protonation step is 5.22 ± 0.02 and is comparable to that calculated by UV-vis spectroscopic studies and was found to be 5.91 ± 0.05 [25]. The overall stability formation constants of complexes [Cu(Hcacp)^−^] and [Cu(H_2_cacp)(H_2_O)] are greater than those of the iminodiacetate copper (II) complexes [Cu(ida)] (log *β* 10.42) and [Cu(H)(ida)] (log *β* 12.35) [33]. The higher stability is ascribable to the coordination of the phenolate oxygen atom. This is also supported by the X-ray crystallographic studies which show that the deprotonated form, even at low pHs, strongly interacts with the metal ion. In addition, the planar configuration of the phenyl ring fixes the orientation of the flexible carboxylate groups in positions favorable to chelating, especially in the case of the copper(II) ion which forms stable complexes in an octahedral/or square pyramidal coordination geometry pattern [36].

One very significant result of this potentiometric titration study is the detection of the dimeric species [Cu_2_(Hcacp)_2_]^2−^. Previous potentiometric studies have postulated that the dimeric complexes are not favored in solution because of steric effects and electrostatic destabilization which do not allow a dimerization process [35]. Harris et al. had suggested the formation of a mononuclear phenolate complex of Cu^2+^ and the phenol iminodiacetate ligand HBIDA at pH above 6.0 (Scheme 1), but they have not mentioned the possibility of dimeric binuclear species in solution [24]. However, recently Stylianou et al. [25] have isolated and crystallographically characterized the dimeric species [Cu_2_(Hcacp)_2_]^2−^ from aqueous solution at alkaline pHs 8.0-9.0, indicating that such species are present in solution. In this complex the two Cu^2+^are bridged through the deprotonated phenolate oxygen (Scheme 2). The speciation diagram of Cu(II)-H_4_cacp system in Figure 3 shows that [Cu_2_(Hcacp)_2_]^2−^ is the major complex at pH range 7.0–8.5 reaching a maximum of 20% of the total metal ion concentration at pH 8.0 and an overall stability formation constant 11.26 ± 0.02 equation (2).


(2)
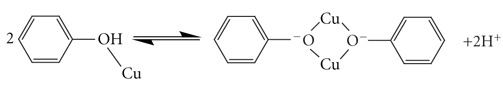



### 3.3. Cu(II)-*H*_6_bicah

**Figure 4:**
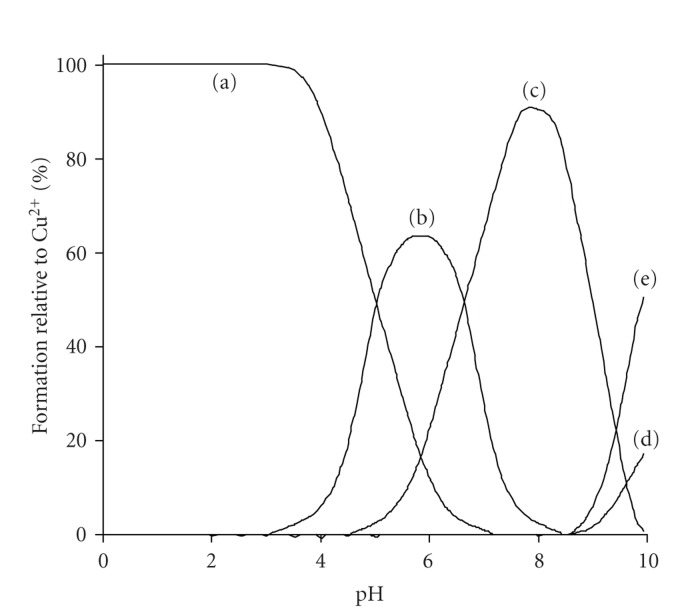
Species distribution (% formation relative to Cu^2+^) in the Cu(II)-H_6_bicah system with metal-to-ligand molar ratio 1 : 2 concentration (H_6_bicah 5.00 mmol dm^−3^) as a function of pH over the range 2.00–10.0 (25°C, *I* = 0.10 mol dm^−3^ KCl, pK_w_ = 13.78, and *γ* = 0.78). The Cu(II) species are as follows: (a) [Cu_2_(H_2_bicah)(H_2_O)_2_], (b) [Cu_2_(Hbicah)(H_2_O)_2_]^−^, (c) [Cu_2_(bicah)(H_2_O)_2_]^2−^, (d) [Cu_2_(bicah)(OH)(H_2_O)]^3−^, and (e) [Cu_2_(bicah)(OH)_2_]^4−^.

The Cu(II)-H_6_bicah titration curves were evaluated on the assumption of the formation of various 1 : 1 and 2 : 1 metal chelates with different protonation steps. The best fit between the simulated curves and the experimental data (Figure 2(b)) was obtained by the speciation model listed in Table 2. Species distribution curves for the complexes formed in the Cu(II)-H_6_bicah system as a function of pH are depicted in Figure 4. In contrast to H_4_cacp, H_6_bicah exhibits two metal binding sites, thus, the ligand may ligate up to two metal ions. The potentiometric study shows that the 1 : 1 species are unstable and the equilibrium is favoured only to the formation of 2 : 1 metal to ligand complexes. In addition, the binucleating ligand, H_6_bicah, exhibits larger steric hindrance than H_4_cacp and thus does not form O_phenolate_-bridged complexes with Cu^2+^ in solution or in solid state. At pH above 9.5 the di- and mono-hydroxo complexes [Cu_2_(bicah)(OH)_2_]^4−^ and [Cu_2_(bicah)(OH)(H_2_O)]^3−^ are the major species with stability formation constants 11.57 ± 0.15 and 15.72 ± 0.11, respectively. The brown [Cu_2_(bicah)(H_2_O)_2_]^2−^ is the major species between pH 7.0 and 9.5 and the green monoprotonated [Cu_2_(Hbicah)(H_2_O)_2_]^−^ at pH range 5.0 to 7.0. The second phenol is protonated at pH below 5.0 resulting in the formation of the blue neutral [Cu_2_(H_2_bicah)(H_2_O)_2_] which has been previously characterized by single crystal X-ray crystallography (Scheme 2) [25]. The two pK_*a*_values for the two equilibriums of the stepwise protonation of the two phenolate oxygen atoms equation (3) have been calculated as 5.89 ± 0.10 and 6.43 ± 0.10 for p*K*_a1_ and pK_*a*2_, respectively. These values are close to the values 6.25 ± 0.08 and 7.19 ± 0.08 for pK_*a*1_ and pK_*a*2_, respectively, found by spectrophotometric studies [25]. These differences are observed because the model used for the calculations in the spectrophotometric studies was incomplete (only the equilibriums in (3) were taken into account):


(3)
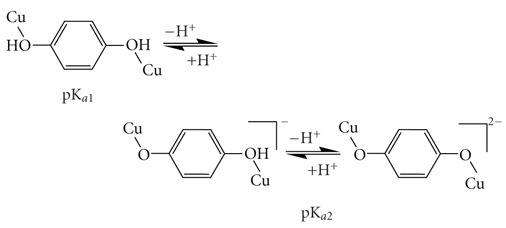



The fact that there is almost 0.5 pK unit difference between the two deprotonation steps indicates that the electronic interaction between the two metal centres through the hydroquinone bridge is significant.

A comparison between the overall stability constants of the two ligands in this study shows that the bifunctional ligand H_6_bicah forms more stable complexes than H_4_cacp in solution. This extra stabilization is attributed to the larger increase of entropy expected for the formation of the binuclear Cu^2+^-H_6_bicah complexes compared to the mononuclear Cu^2+^-H_4_cacp.

## 4. Conclusions

The speciation of Cu^2+^ with the iminodiacetic phenol/hydroquinone ligands H_4_cacp/H_6_bicah in aqueous solution was investigated by pH-potentiometry. Ligand H_4_cacp, at pH below 5.0 forms with Cu^2+^ the mononuclear 1 : 1 and 1 : 2 complexes. At higher pH the phenol proton is deprotonated and at pH range 5.0–7.0 the major species is the mononuclear 1 : 1 complex. However at pH 7.0-8.0 the formation of a binuclear complex takes place and it is attributed to a O_phenolate_-bridged complex. The binucleating ligand H_6_bicah forms only 2 : 1 metal to ligand complexes in the pH range 2.0 to 9.0. The major species are the complete phenol protonated complex at pH below 4.5, the monoprotonated at pH range 4.5 to 7.0, and the complete phenol deprotonated species between pHs 7.0 and 9.0. The H_6_bicah did not form binuclear O_phenolate_-bridged complex in solution probably due to steric hindrance originated from the binucleating nature of the ligand. On the other hand, this solution study shows that binuclear O_phenolate_-bridged species must also be considered in speciation studies of Cu^2+^ ions with mononucleating phenolate ligands such as H_4_cacp.

## Figures and Tables

**Table 1:** Compositions, overall stability formation constants (log *β*), and acidity constants (pK_*a*_) for the species formed in *H*_4_cacp and Cu(II)-*H*_4_cacp system, over the pH range 2.00–10.0 thus obtained from the potentiometric study (25°C, *I* = 0.10 *m* *o* *l* *d* *m* ^−3^ *K* *C* *l*, pK_*w*_ = 13.78, and *γ* = 0.78).

(*p*, *q*, *r*)	Species	log *β*	pK_*a*_
(0, 1, 1)	[H_2_cacp]^2−^	8.40 ± 0.01	8.47^a^
(0, 1, 2)	[H_3_cacp]^−^	13.18 ± 0.04	4.84^b^
(0, 1, 3)	[H_4_cacp]	15.56 ± 0.02	2.42^c^
(1, 1, −1)	[Cu(Hcacp)(OH)]^2−^	8.17 ± 0.01	
(2, 2, 0)	[Cu_2_(Hcacp)_2_]^2−^	11.26 ± 0.02	
(1, 1, 0)	[Cu(Hcacp)(H_2_O)]^−^	14.58 ± 0.02	
(1, 2, 2)	[Cu(H_2_cacp)_2_]^2−^	17.62 ± 0.02	
(1, 1, 1)	[Cu(H_2_cacp)(H_2_O)]	22.94 ± 0.01	

^a^Phenolate or aromatic carboxylate oxygen group, ^b^carboxylate oxygen group, ^c^amine nitrogen group.

**Table 2:** Compositions, overall stability formation constants (log *β*), and acidity constants (pK_*a*_) for the species formed in *H*_6_bicah and Cu(II)-*H*_6_bicah system, over the pH range 2.00–10.0 thus obtained from the potentiometric study (25°C, *I* = 0.10 mol *d* *m* ^−3^ KCl, p*K*_*w*_ = 13.78, and *γ* = 0.78).

(*p*, *q*, *r*)	Species	log *β*	pK_*a*_
(0, 1, 0)	[H_2_bicah]^4−^	8.41 ± 0.02	8.47^a^
(0, 1, 2)	[H_4_bicah]^2−^	13.40 ± 0.01	7.26^b^
(2, 1, −2)	[Cu_2_(bicah)(OH)_2_]^4−^	11.57 ± 0.15	
(2, 1, −1)	[Cu_2_(bicah)(H_2_O)(OH)]^3−^	15.72 ± 0.11	
(2, 1, 0)	[Cu_2_(bicah)(H_2_O)_2_]^2−^	32.90 ± 0.16	
(2, 1, 1)	[Cu_2_(Hbicah)(H_2_O)_2_]^−^	39.33 ± 0.16	
(2, 1, 2)	[Cu_2_(H_2_bicah)(H_2_O)_2_]	45.52 ± 0.12	

^a^ Phenolate oxygen group, ^b^ carboxylate oxygen group.
